# Serologic Evidence of Widespread Everglades Virus Activity in Dogs, Florida

**DOI:** 10.3201/eid1212.060446

**Published:** 2006-12

**Authors:** Lark L. Coffey, Cynda Crawford, James Dee, Ryan Miller, Jerome Freier, Scott C. Weaver

**Affiliations:** * University of Texas Medical Branch, Galveston, Texas, USA;; † University of Florida, Gainesville, Florida, USA;; ‡ Hollywood Animal Hospital, Hollywood, Florida, USA;; § Animal and Plant Health Inspection Service, Fort Collins, Colorado, USA

**Keywords:** Everglades virus, epidemiology, serosurvey, Venezuelan equine encephalitis virus, ecology, research

## Abstract

Everglades virus (EVEV), an alphavirus in the Venezuelan equine encephalitis complex, circulates among rodents and vector mosquitoes in Florida and occasionally infects humans. It causes febrile disease, sometimes accompanied by neurologic manifestations. Although previous surveys showed high seroprevalence in humans, EVEV infections may be underdiagnosed because the disease is not severe enough to warrant a clinic visit or the undifferentiated presentations complicate diagnosis. Documented EVEV activity, as recent as 1993, was limited to south Florida. Using dogs as sentinels, a serosurvey was conducted to evaluate whether EVEV circulated recently in Florida and whether EVEV's spatial distribution parallels that of the mosquito vector, *Culex cedecei*. Four percent of dog sera contained neutralizing EVEV antibodies, and many seropositive animals lived farther north than both recorded EVEV activity and the principal vector. These results indicate that EVEV is widespread in Florida and may be an important, unrecognized cause of human illness.

Everglades virus (EVEV), a mosquitoborne Venezuelan equine encephalitis (VEE) complex alphavirus (Togaviridae; Alphavirus), circulates continuously in enzootic foci in Florida. EVEV infection of humans can result in a nonspecific, flulike, febrile illness that can progress to severe neurologic disease (*1**,**2*). Human EVEV serosurveys in the 1960s and 1970s indicated that people in south Florida were frequently exposed to EVEV. In 1 survey (*3*), >50% of Seminole Indians who resided north of Everglades National Park had antibody to EVEV, and 9% of other groups living in 3 rural communities at the periphery of the park were EVEV seropositive in 1973 (*4*).

Despite high antibody prevalence, most seropositive persons reported no history of symptoms or signs consistent with VEE-like disease, although exceptions have been noted (*1**,**2*). Among the small number of seropositive persons who experienced illnesses consistent with EVEV infection, the most common signs and symptoms were fever, myalgia, headache, tender lymph nodes, and diarrhea (*4*). Although serologic data indicate that persons are frequently exposed to EVEV, disease is probably most often asymptomatic or not sufficiently severe to require a visit to a physician. In addition, if an EVEV-infected person seeks medical attention, the nonspecific clinical signs and symptoms, similar to those caused by other viral diseases, may not warrant etiologic diagnosis. Persons with undiagnosed diseases of suspected viral etiology are not routinely tested for EVEV by the Florida Department of Health (L.M. Stark, pers. comm.). However, repeated evidence of EVEV antibody in persons living at different locales in south Florida (*3**,**4*) suggests that EVEV may be an unrecognized cause of febrile illness.

All recorded EVEV activity has been limited to south Florida, from Everglades National Park north to Indian River County (*5*) (Figure 1). The last EVEV isolation was reported in 1993 (*6*). As is the case with surveillance for many arboviruses, EVEV activity may be noted only in regions where virologists actively search. No recent serosurveys to detect current EVEV transmission have been performed, and the geographic distribution of EVEV circulation has never been defined by comprehensive surveys. Laboratory susceptibility experiments suggest that Culex (Melanoconion) cedecei may be the only important EVEV vector (*7**,**8*). This species has only been reported in 12 southern counties of Florida (*9*), which indicates that if the mosquito vector regulates EVEV distribution, EVEV activity is probably limited to those areas. Therefore, the goal of this study was to answer 3 questions: 1) Has EVEV recently been circulating in Florida? 2) What is the geographic distribution of EVEV throughout the state? and 3) Does the spatial distribution of EVEV activity, as measured by seroprevalence, parallel the recorded distribution of the principal vector, Cx. cedecei?

**Figure 1:**
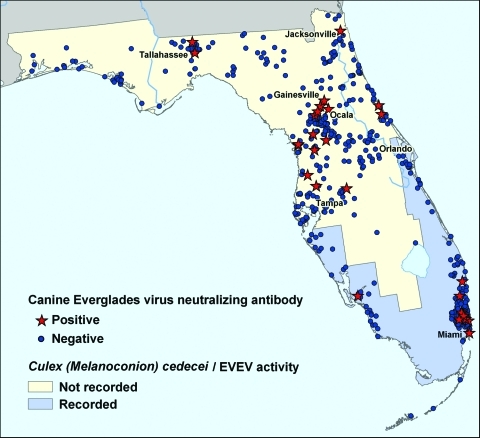
Everglades virus–seropositive and -seronegative dogs in Florida, 2003–2004. A total of 633 samples of dog sera from the Veterinary Medical Center in Gainesville or Hollywood Animal Hospital in Miami were screened. Each blue dot (seronegative) or red star (seropositive) represents a single dog. Most of the seropositive dogs lived in north-central Florida, outside the recorded range of the principal vector *Culex* (*Melanoconion*) *cedecei* or previously recorded Everglades virus activity (purple shading). Owners of dogs living outside the endemic region reported that their animal had not traveled to south Florida.

Testing human sera for EVEV antibody would be an ideal measure of human exposure, but obtaining these samples is difficult from a logistic and regulatory standpoint. Some human pathogen studies have used antibody prevalence of domestic animals to predict human disease risk (*10**–**12*). Canines are effective EVEV sentinels; hemagglutination-inhibiting (HI) and neutralizing antibodies without clinical disease developed in military sentry dogs stationed outdoors in Homestead, Florida (*13*). Dogs experimentally infected with VEEV (strains not reported) survived infection, and minimal HI titers of 320 developed (*14*). Furthermore, beagles exposed to Aedes triseriatus mosquitoes infected with the VEEV subtype IAB Trinidad Donkey strain became viremic from days 1 to 5 postinoculation, with virus titers ranging from 1 to 3.8 log_10_ mouse intraperitoneal median lethal doses per milliliter (MIPLD_50_) (*15*). In a reciprocal study, experimentally infected beagles with viremias of at least 3.7 log_10_ MIPLD_50_ of VEEV were capable of infecting Ae. triseriatus mosquitoes (*16*). Taken together, these results demonstrate that dogs become infected with EVEV or VEEV by artificial and natural inoculation routes; produce viremias of 3–4 days' duration; sustain a nonfatal, febrile infection; and develop detectable antibody.

Field studies in VEEV-enzootic foci outside Florida also indicate that dogs are frequently infected during outbreaks. Domestic dogs tested after epidemics in Colombia (*17*), Venezuela (*18*), and Guatemala (*19*) commonly had neutralizing antibody to VEEV. In these dogs, the average neutralizing antibody titer was lower than titers in experimentally infected canines, possibly reflecting a longer window of time between exposure to VEEV and the time of blood collection, a period during which antibody levels could wane.

Because pet dogs live in close proximity to humans and can serve as effective EVEV sentinels, human exposure to EVEV can be estimated on the basis of dog seroprevalence. Pet dogs are also good sentinels for human arbovirus risk because they more closely approximate the biomass of a human than a hamster or another small mammal, they are restricted to confined geographic zones such as a backyard or neighborhood, and dog owners are knowledgeable about the travel history of their pets. Therefore, we evaluated the distribution of EVEV in Florida by using pet dogs as sentinels of EVEV activity.

## Materials and Methods

### Serum Collection

Whole blood was collected from pet dogs seen for treatment of various conditions at the University of Florida Veterinary Medical Center in Gainesville, Florida, USA, from July 2003 to January 2004, and at Hollywood Animal Hospital in Hollywood (Miami), Florida, from June to December 2004. Samples from animals living far from Florida, in areas not known to be enzootic for VEEV complex alphaviruses (Galveston, Texas, USA, and Munich, Germany) were kindly provided by resident veterinarians at local clinics and used as negative controls. Dogs from all locations were randomly sampled independent of the reason for the clinic visit. Serum was separated from erythrocytes after low-speed centrifugation. For dogs seen in Gainesville, each pet owner was asked whether the animal had traveled outside of their city of residence, except for the visit to the veterinary clinic.

### Antibody Assays

Each serum sample was tested by standard 80% plaque reduction neutralization test (PRNT) (*20*). In brief, neutralizing antibody titers were determined by a constant-virus, serum dilution procedure that used Vero (African green monkey kidney) cell monolayers attached to 6- or 12-well plates. Serum samples were heated at 56°C for 30 min for inactivation, and a 1:10 starting serum dilution was serially 2-fold diluted and incubated with an equal volume (250 μL) of ≈800 PFU/mL of EVEV strain FE3–7c for 1 h at 37°C. The virus-serum mixture was incubated onto confluent Vero cell monolayers, overlaid after 1 h with 0.4% agar in Eagle's minimal essential medium and incubated at 37°C for 2 days. The virus was inactivated with 10% formaldehyde, and cell monolayers were stained with 0.05% crystal violet in 30% methanol to visualize plaques. Dilutions of serum that caused a >80% reduction in the number of plaques, as compared with negative controls (commercial fetal bovine serum and serum from dogs living outside alphavirus-enzootic areas [Texas and Germany]), were considered positive. Serum from an experimentally infected, EVEV-immune cotton rat was included as a positive control. The reciprocal of the highest dilution of serum (indicated as the final virus-serum dilution) that inhibited at least 80% of plaques was recorded as the antibody titer. To rule out infection with Eastern equine encephalitis virus (EEEV) and Highlands J viruses (HJV), related alphaviruses also enzootic to Florida, all EVEV-seropositive sera were screened by PRNT for antibody to these viruses by using the North American EEEV strain FL-93 (*21*) and HJV strain 86–31227 (*22*).

### Location Mapping

To delineate the geographic distribution of antibody to EVEV in canines, the home location of each dog was mapped with ArcGIS for ArcView 9.1 (ESRI, Redlands, CA, USA). Owner street addresses were geocoded (Tele Atlas, Lebanon, NH, USA) and overlaid with land cover and water features (e.g., lakes, ponds, reservoirs, streams, wetlands) from the National Land Cover Dataset (United States Geological Survey, EROS Data Center, Sioux Falls, SD, USA). The proximity of each dog residence relative to water features and forest, agricultural, or suburban or urban land was calculated. The landcover type at the residence location was also determined.

## Results

### Serology

A total of 633 serum samples from dogs living in Florida were tested for EVEV antibody by PRNT. Of these, 422 were from the Gainesville clinic, and 211 were from the Miami clinic. At least 1 serum sample was obtained from 54 of the 67 counties in Florida, and >20 samples per county were tested from 6 counties, including the greater Miami-Dade area (Broward n = 152, Miami-Dade n = 55, and Palm Beach n = 27) and Ocala region (Marion n = 63). Although the greatest number of samples came from the Miami area, the relative number of dog sera collected per human population by county was highest for Marion County (1 dog per 4,330 people), compared with ratios of 1:45,392 and 1:12,544 in Miami-Dade and Broward Counties, respectively.

Of the 633 sera tested, 26 (4%) contained antibody to EVEV, with 80% PRNT endpoint titers ranging from 20 to 2,560 (Table). None of the EVEV PRNT-positive sera contained detectable neutralizing antibody against EEEV or HJV, which ruled out the possibility of cross-reactions with related, sympatric alphaviruses. None of the dogs from Texas or Germany had detectable antibody to EVEV. The proportion of 80% PRNT-seropositive dogs from Florida (26/633) was significantly greater than that for dogs from Texas and Germany (0/61),which indicated that serosurvey results from Florida dogs were not false positives (χ^2^ = 2.6, degrees of freedom [df] = 1, p<0.05).

**Table:** Endpoint plaque reduction neutralization test titers for Everglades virus–seropositive dogs from Florida*†

Approximate age (mo)	80% EVEV PRNT titer	Travel history to south Florida
108	320	No
56	40	No
90	20	No
66	40	No
119	2,560	Resident Miami
67	20	Resident Miami
159	20	Resident Miami
129	160	Resident Miami
9	40	Resident Miami
76	80	Resident Miami
80	20	Resident Miami
130	80	No
121	40	No
78	80	No
80	80	No
140	80	No
62	20	No
132	20	No
86	40	No
58	80	No
62	20	No
101	20	No
114	80	No
95	20	No
114	40	No
18	20	No

*EVEV, Everglades virus; PRNT, plaque reduction neutralization test. †In addition, an experimentally infected cotton rat had a 5,120 80% EVEV PRNT titer.

Most of the EVEV-seropositive dogs had PRNT endpoint titers in the low range (20–40). Although the 80% EVEV PRNT titer for 38% of the 26 seropositive dogs was only 20, this value is considered protective for humans vaccinated with the experimental VEEV vaccine and is comparable to levels observed in dogs after outbreaks in Venezuela (*18*). The youngest EVEV antibody–seropositive dog was born in 2003, indicating that EVEV has been circulating recently in the Miami-Dade area.

### Geographic EVEV Distribution

A map of the geographic distribution of antibody to EVEV in canines in Florida showed that EVEV-seropositive dogs were spatially dispersed throughout the state (Figure 1). The seropositive dogs resided in major human population centers (Miami, Ocala, and Tallahassee), areas from which more samples were collected because this study focused on domestic canines. Unexpectedly, we observed that EVEV-seropositive dogs lived farther north than the recorded EVEV distribution, which only extends to Indian River County (*9*). Owners of all 16 seropositive dogs from northern and central Florida reported that their pet had not traveled to counties in southern Florida, where EVEV had been previously recorded. The reported Cx. (Mel.) cedecei distribution extends only as far north as Brevard County (*9*). The 633 serum samples were grouped on the basis of counties in which EVEV activity or Cx. cedecei have been previously recorded (Figure 1). A total of 286 samples were from dogs in counties in which the mosquito vector or EVEV has been recorded ("recorded"), and 347 samples were from dogs in counties where neither the vector nor virus has been recorded ("not recorded"). No significant differences in seroprevalence were found between the recorded group (9/277) and the not-recorded group (18/329) (χ^2^ = 1.59, df = 1, p>0.05).

### Environmental Characteristics of Seropositive-Dog Residences

**Figure 2:**
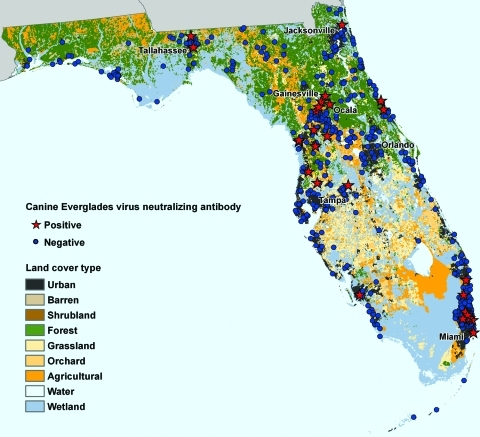
Residence locations of Everglades virus–seropositive and –seronegative dogs in Florida and landcover habitat types obtained from the National Land Cover Database.

Figure 2 shows residence locations of EVEV-seropositive dogs overlaid on Florida National Land Cover Data (NLCD) types. Satellite imagery showed that EVEV-seropositive dogs were no more likely to live in rural, forested, or agricultural areas favored by Cx. (Mel.) cedecei than seronegative animals (data not shown). Most (16/26) EVEV-seropositive dogs lived in suburban, urban, or residential environments, a proportion no different from the proportion found for 26 EVEV-seronegative dogs (16/26) chosen by random selection. NLCD data are generally used for broad-scale analysis and do not classify microhabitats. These observations indicate that microhabitats may play a more important role as a predictor of EVEV canine seropositivity than NLCD classifications. In addition to habitat type, no association was found between the location of EVEV-seropositive dogs and average annual precipitation, average annual or minimum temperature, and proximity to bodies of water (data not shown).

## Discussion

The objective of this study was to determine the distribution of EVEV activity in Florida on the basis of seroprevalence in pet dogs that live in close proximity to humans. Although virus isolation from mosquitoes or vertebrates would be the most definitive measure of virus activity, attempting to isolate EVEV throughout Florida is logistically challenging and not currently conducted by the state health department. To more precisely determine locations in which canine EVEV infection occurs, seronegative dogs could be housed in specific habitat types in which mosquitoes test positive for EVEV. Other studies that would be useful to corroborate our findings include serosurveys in rodents that serve as EVEV reservoir hosts and that have very limited dispersal ranges. Cattle with known histories have also proven useful as VEEV sentinels (*23*).

### EVEV Seropositivity in Northern Florida

Detection of EVEV antibody in dogs living in north and central Florida, without history of travel to south Florida, where EVEV was previously known to be endemic, indicates that EVEV distribution probably extends farther north than previously reported. Possible explanations for this unexpected finding include the following: 1) Dogs residing in the northern parts of Florida became infected during their trip to the veterinary clinic in Gainesville (a clinic visit was common to the history of all of these dogs). We believe that this explanation is highly unlikely because the clinics are indoors and probably are not infested by many mosquitoes and because even if a dog were bitten by an infected mosquito in or on the way into the clinic, dogs do not seroconvert until several days after infection. 2) Cross-reactive antibodies generated by other alphaviruses endemic to northern Florida were not responsible for EVEV neutralizing antibodies. Overall, 4% of the dog sera we tested contained neutralizing EVEV antibodies, a rate similar to the seroprevalence for the major human pathogenic arboviruses (EVEV, EEEV, Saint Louis encephalitis virus, Western equine encephalitis virus) in Florida detected in >2,500 sera from wildlife in 38 of the state's 67 counties from 1965 to 1974 (*24*). However, alphavirus-neutralizing antibodies generally do not cross-react, and none of the EVEV-seropositive dogs had neutralizing antibodies to EEEV or HJV, which indicates that the dogs sampled were not infected with any known, related, sympatric alphaviruses.

When one considers that pet dogs live close to humans and experience similar mosquito exposure as standard arbovirus sentinels that are housed outdoors continuously in cages (e.g., hamsters, chickens), these results suggest that human infections with EVEV may also occur regularly in many areas of Florida. Therefore, implementing procedures to screen for EVEV in cases of febrile illness or encephalitis might result in recognition of undiagnosed disease.

Our geographic results may have been skewed by the fact that two thirds of the dogs sampled were taken to the referral hospital in Gainesville, potentially oversampling dogs from this region of the state. Despite limitations inherent to using serum samples that were not randomly collected from all areas in Florida, detection of antibody in north and central Florida suggests that the geographic distribution of EVEV is more extensive than previously recorded and extends as far north as Tallahassee. EVEV distribution could also extend outside of Florida, although a serosurvey of raccoons in Georgia did not show EVEV activity that far north (*25*).

### Determinants of EVEV Distribution

These results raise a question: What determines the spatial EVEV distribution in Florida? Several hypotheses warrant consideration. First, reservoir host susceptibility and viremia limit the EVEV distribution. The distribution of different cotton rat populations with respect to the known EVEV distribution has not been delineated. However, genetically distinct cotton rats from outside Florida are highly permissive for EVEV viremia (*26*), which diminishes support for this theory. Second, mosquito species other than Cx. (Mel.) cedecei serve as enzootic vectors in northern Florida. The most abundant mammalophilic species in regions of Florida in which EVEV was previously detected, Aedes taeniorhynchus and Cx. nigripalpus, are not competent EVEV vectors in laboratory experiments (*8*). Although marginally susceptible mosquito species have been implicated as arbovirus vectors during outbreaks when their large population sizes allow for efficient transmission, the species implicated in those settings showed at least some competence in laboratory susceptibility experiments (*27**,**28*). By contrast, Ae. taeniorhynchus and Cx. nigripalpus were completely refractory to experimental laboratory infection with EVEV (*8*). Also, enzootic viruses in the VEEV complex, including EVEV, are typically highly specific in their use of Culex. (Melanoconion) spp. as primary vectors (*29*). Of the 7 vectors of enzootic VEE complex viruses identified to date, all are members of the Spissipes section in the Culex (Melanoconion) subgenus (*29*). Nevertheless, other species, as well as populations of Ae. taeniorhynchus and Cx. nigripalpus from northern Florida, should be evaluated in laboratory studies. Field studies should also be conducted since EVEV circulating in northern Florida could be capable of infecting Ae. taeniorhynchus or Cx. nigripalpus, unlike EVEV isolates and mosquitoes from the Everglades that were used for laboratory vector competence studies. Ticks or other ectoparasites that are widely distributed in Florida could also serve as vectors. Finally, one other possible explanation is that the distribution of EVEV is limited by that of Cx. cedecei, but the range of this vector extends beyond that previously recorded or has expanded (or both). Because this species is difficult to identify morphologically, systematic mosquito sampling throughout the state is needed to address this possibility.

Location mapping showed that EVEV-seropositive dogs were not more likely than seronegative dogs to live in environments typically inhabited by Cx. cedecei. These results should be interpreted with caution because of limitations inherent to using owner reports of pet travel histories. Cx. cedecei has been trapped in hardwood hammocks in the Everglades, mangrove swamps, and hardwood forests, but little is known about its proclivity for disturbed or suburban habitats. A closely related VEEV complex virus vector that occurs in Central America, Cx. (Mel.) taeniopus (*30*), is found in high abundance in habitats subject to heavy human disturbance. Cx. cedecei might also inhabit suburban or urban areas, but it has not been identified in such environments because of limitations in identification practices.

### Clinical Data

In spite of antibody detection in 4% of animals sampled, clinical signs reported for all of the seropositive dogs that were seen at the referral veterinary hospital in Gainesville (detailed data not shown; most animals were diagnosed with tumors or bacterial infections) were inconsistent with EVEV disease in humans (*2*) or laboratory rodents (L.L. Coffey, unpub. data). These observations indicate that EVEV was likely not the underlying cause for the manifestations seen at these hospitals. Military sentry dogs observed continuously during periods of natural infection, verified by seroconversion, did not show signs of VEE-like illness (*13*).

Despite the low EVEV antibody titers observed in many of the EVEV-seropositive dogs, these results likely represent real infections, especially given that no serum samples from 61 dogs living in areas outside Florida tested positive. Antibody titers in sentry dogs naturally infected with EVEV waned to low levels during a 1- to 2-year period (*13*). The low titers reported here could result from a long interval between infection and blood collection. Alternatively, high antibody titers may never develop in dogs naturally infected with EVEV. For example, 1 young dog born in September 2003 and then sampled <1 year later had a titer of only 40 (Table). The low EVEV antibody titers in naturally infected pet dogs in this study are similar to those in sentry dogs (*13*) but are not as high as titers in dogs experimentally injected with 1,000 suckling mouse ICLD_50_ of EVEV (*31*) or VEE virus (*14*). Higher neutralizing antibody titers might have developed in experimentally infected dogs because the dose of virus administered by intramuscular injection was greater than the amount delivered by a feeding mosquito.

In summary, detection of antibody in dogs throughout Florida suggests that EVEV extends as far north as Tallahassee and has been circulating as recently as 2003. Additional serosurveys involving more dogs, rodents, or both throughout the state, in addition to attempts to isolate EVEV from mosquitoes and vertebrates in regions where seropositive dogs occur, will further define the geographic distribution of EVEV. Enhanced vector surveillance could better define the range of Cx. cedecei and may help to explain the unexpected finding of EVEV activity in northern Florida. Screening for EVEV in human patients may also show wide spatial dispersion and a high rate of human infection. EVEV may be a cause of febrile illness or encephalitis in many areas of Florida and should be considered by physicians as a potential cause.
